# Uncertainty should be the rule, not the exception: Integrating fibre orientation variability in cardiac electrophysiology modelling

**DOI:** 10.1113/JP289737

**Published:** 2025-09-04

**Authors:** Ovais Ahmed Jaffery, Caroline Helen Roney

**Affiliations:** ^1^ School of Engineering and Materials Science Queen Mary University of London London UK

**Keywords:** cardiac electrophysiology, uncertainty quantification, ventricular modelling

Computational modelling of cardiac electrophysiology has significantly advanced in recent years, with models exhibiting increasing anatomical detail, personalized inputs and physiological sophistication. However, many of these models rely on parameters derived from previously published literature, often reflecting specific experimental measurements or population averages, and may either assume that personalized inputs are precisely known or require them to be estimated through calibration. In reality, every parameter in cardiac modelling – from cellular parameters to fibre orientation – is subject to variability, measurement error and biological heterogeneity. Despite this, systematic uncertainty quantification (UQ) to evaluate the effects of parameter variability on forward model outputs is not typically implemented in cardiac modelling studies. Tanner et al. (2026) seek to change this by presenting a methodology for UQ applied to the effects of fibre orientation variability on ventricular activation. Using a full‐ventricle model and an eikonal‐based approach, they applied UQ methods to assess how physiological variability in fibre orientation influences cardiac activation patterns. They demonstrate that UQ is both useful and feasible at the organ scale.

Fibre architecture governs the direction and velocity of activation wavefronts in cardiac tissue and is important for investigating arrhythmia mechanisms. Despite this importance, many ventricular modelling studies rely on rule‐based algorithms to assign a smooth transmural rotation, assuming that a linear transition from epicardium to endocardium is representative across individuals. This modelling assumption does not capture variability between patients or allow personalization. Image‐based approaches, such as diffusion tensor magnetic resonance imaging in a beating heart, are in an early phase of research and rarely available. Moreover, even when suitable imaging data are available, the effects of measurement uncertainty and structural heterogeneity on model predictions must still be accounted for to assess a measure of credibility associated with those predictions.

UQ offers a paradigm to model fibre orientations not as a fixed input, but as a distribution with physiological variability. Utilizing a range of UQ techniques, simulation input parameters are modelled as random variables with a probability distribution reflecting parameter variation. This randomness is pushed forward to the model output, resulting in a random activation time field. The output distribution is then analysed using UQ computational techniques to approximate statistics including mean and standard deviation, and a global sensitivity analysis may be performed.

Tanner et al. (2026) employed a simplified framework to model a healthy heart with ectopic pacing, selecting a controlled substrate to isolate the effects of fibre orientation variability and to demonstrate UQ in a reproducible and interpretable setting. This represents a baseline from which more complex scenarios including infarcts and ventricular arrhythmias can be considered. Spatial heterogeneity in sensitivity to the alpha angle, the inclination of fibre rotation, highlights that output metrics and activation patterns need to be examined in three dimensions and not simplified to a scalar metric.

Tanner et al. (2026) used eikonal solvers for forward simulations, selected for computational efficiency. Eikonal solvers capture activation (depolarization) patterns only. The current eikonal model does not explicitly include the His–Purkinje system, which can significantly impact activation sequences. Future work should investigate UQ analysis using more complex models, including the monodomain and bidomain models, in the setting of varied anisotropy, rate dependence, repolarization dynamics and re‐entry mechanisms. Extensions to consider the effects of fibre orientation on cardiac electromechanics are also important.

UQ can be performed using various methodologies. One of the simplest is Monte Carlo, which randomly samples the parameter space but requires extensive simulations. More efficient techniques include surrogate models (emulators) that approximate the real model output. Example emulators include Gaussian process emulation (GPE) and polynomial chaos expansion (PCE). Tanner et al. (2026) used PCE because it efficiently quantifies uncertainty with a smaller number of simulations. PCE is well suited for problems where input uncertainties are represented by probability distributions, allowing the output response to be approximated with orthogonal polynomials to yield statistical moments and sensitivity indices.

GPEs are powerful but can be computationally expensive to train. GPEs have been used for global sensitivity analysis in four‐chamber heart computational fluid dynamics (Karabelas et al., 2022), electromechanics, and restitution curve emulators (Coveney et al., 2021). Paun et al. (2025) compared GPE and PCE for haemodynamic pulse‐wave propagation modelling, showing GPE slightly outperformed PCE in pulmonary circulation modelling. We recommend a similar comparison study for cardiac electrophysiology modelling, using open‐source toolboxes for GPE (https://github.com/SheffieldML/GPy) and PCE (Narayan et al., 2023).

In clinical translation, deterministi models can convey a misleading sense of certainty. For example, using simulations to guide ablation therapy or to localize premature ventricular contractions assumes that activation sequences are repeatable and predictable. However, if outputs vary significantly with small changes in fibre orientation, conductivity or anatomy, deterministic results may mislead rather than provide mechanistic insights. Uncertainty must be considered to transform deterministic models to reliable computational tools that can be adopted and trusted in the clinic.

In the future, UQ should be embedded in cardiac modelling. Tanner et al. (2026) provide an example of applying UQ in a simple setting with the vision of extending to more complex applications. The cardiac computational community is encouraged to use this approach for all uncertain inputs, from subcellular and cellular parameters to tissue‐scale conductivities and observable features such as anatomy, pacing sites, and endocardial or body surface electrical potentials. Sensitivity analysis and UQ are vital for interpreting predictions, and acknowledgment of input uncertainty should become a standard practice. Tanner et al. (2026) demonstrate that UQ is not only computationally feasible but essential for building clinically meaningful and mechanistically grounded cardiac models. Their open‐source software tool, UncertainSCI (Narayan et al., 2023), enables UQ analysis in cardiac digital twin studies.

## Additional information

### Competing interests

No competing interests declared.

### Author contributions

O.J.: Conception or design of the work; Drafting the work or revising it critically for important intellectual content; Final approval of the version to be published; Agreement to be accountable for all aspects of the work. C.R.: Conception or design of the work; Drafting the work or revising it critically for important intellectual content; Final approval of the version to be published; Agreement to be accountable for all aspects of the work.

### Funding

UKRI | Medical Research Council (MRC): Caroline H. Roney, MR/W004720/1 This research was funded by a UKRI Future Leaders Fellowship (MR/W004720/1).

## Supporting information


Peer Review History
