# Supplementary material for: Uncertainty should be the rule, not the exception: Integrating fibre orientation variability in cardiac electrophysiology modelling
Source: J Physiol. 2025 Sep 4;604(13):5080–1. doi: 10.1113/JP289737 (PMC13327751; doi:10.1113/JP289737)
Supplement: Supplementary file 1 — Peer Review History [file TJP-604-5080-s001.pdf]

The Journal of Physiology

Uncertainty Should Be the Rule, Not the Exception: Integrating Fiber Orientation Variability in Cardiac Electrophysiology Modelling

Ovais Jaffery and Caroline H Roney
DOI: 10.1113/JP289737

Corresponding author(s): Caroline Roney (c.roney@qmul.ac.uk)

The referees have opted to remain anonymous.

**Review Timeline:**	Submission Date:	01-Aug-2025
	Accepted:	20-Aug-2025

Senior Editor: Bjorn Knollmann

Reviewing Editor: Viviane Timmermann

Transaction Report:

(Note: With the exception of the correction of typographical or spelling errors that could be a source of ambiguity, letters and reports are not edited. Depending on transfer agreements, referee reports obtained elsewhere may or may not be included in this compilation. Referee reports are anonymous unless the Referee chooses to sign their reports.)

1st Editorial Decision **20-Aug-2025**

Dear Dr Roney,

Re: JP-P-2025-289737 "**Uncertainty Should Be the Rule, Not the Exception: Integrating Fiber Orientation Variability in Cardiac Electrophysiology Modelling** " by Ovais Jaffery and Caroline H Roney

We are pleased to tell you that your paper has been accepted for publication in The Journal of Physiology.

The last Word (or similar) version of the manuscript provided will be used by the Production Editor to prepare your proof. When this is ready you will receive an email containing a link to Wiley's Online Proofing System. The proof should be thoroughly checked and corrected as promptly as possible.

Authors should note that it is too late at this point to offer corrections prior to proofreading. Major corrections at proof stage, such as changes to figures, will be referred to the Editors for approval before they can be incorporated. Only minor changes, such as to style and consistency, should be made at proof stage. Changes that need to be made after proof stage will usually require a formal correction notice.
All queries at proof stage should be sent to: TJP@wiley.com.

Following copyediting and typesetting the accepted version will be published online.

Yours sincerely,

Bjorn Knollmann
Senior Editor
The Journal of Physiology

IMPORTANT POINTS TO NOTE FOLLOWING YOUR PAPER'S ACCEPTANCE:
If you would like to receive our 'Research Roundup', a monthly newsletter highlighting the cutting-edge research published in The Physiological Society's family of journals (The Journal of Physiology, Experimental Physiology, Physiological Reports, The Journal of Nutritional Physiology, and The Journal of Precision Medicine: Health and Disease), please click this link, fill in your name and email address and select 'Research Roundup':
https://www.physoc.org/journals-and-media/membernews

• TRANSPARENT PEER REVIEW POLICY: To improve the transparency of its peer review process, The Journal of Physiology publishes online as supporting information the peer review history of all articles accepted for publication. Readers will have access to decision letters, including Editors' comments and referee reports, for each version of the manuscript as well as any author responses to peer review comments. Referees can decide whether or not they wish to be named on the peer review history document.

• You can help your research get the attention it deserves! Check out Wiley's free Promotion Guide for best-practice recommendations for promoting your work at: www.wileyauthors.com/eeo/guide. You can learn more about Wiley Editing Services which offers professional video, design, and writing services to create shareable video abstracts, infographics, conference posters, lay summaries, and research news stories for your research at: www.wileyauthors.com/eeo/promotion.

• IMPORTANT NOTICE ABOUT OPEN ACCESS: To assist authors whose funding agencies mandate public access to published research findings sooner than 12 months after publication, The Journal of Physiology allows authors to pay an Open Access (OA) fee to have their papers made freely available immediately on publication.

The Corresponding Author will receive an email from Wiley with details on how to register or log-in to Wiley Authors Services where you will be able to place an order

• You can check if your funder or institution has a Wiley Open Access Account here: https://authorservices.wiley.com/author-resources/Journal-Authors/licensing-and-open-access/open-access/author-compliance-tool.html.

EDITOR COMMENTS

Reviewing Editor:

Thank you for this very well written perspective that fits nicely into this special collection on integrating mathematical and experimental research in cardiology which also considers the clinical translation of research findings.

Senior Editor:

Thank you for your excellent contribution to the Journal!

REFEREE COMMENTS

Referee #1:

Thank you for this insightful and well-written perspective. The authors clearly and effectively highlight the importance of UQ in cardiac modeling and its role in improving clinical relevance. I appreciate their emphasis on moving beyond deterministic models and thoughtful suggestions for future work, including pathophysiology and surrogate model comparisons. Their framing of UQ as essential, not optional, aligns well with our goals and strengthens the case for its broader adoption in the field.
